# Expression of tumor necrosis factor-alpha converting enzyme and matrix metalloproteinase-3 in proliferated synovium in a patient with synovitis-acne-pustulosis-hyperostosis-osteitis syndrome: a case report

**DOI:** 10.4076/1752-1947-3-9123

**Published:** 2009-09-15

**Authors:** Koichiro Komiya, Harumoto Yamada, Nobuki Terada, Yoshikazu Mizoguchi, Mitsuko Yamada, Masashi Suzuki, Shinichi Kato

**Affiliations:** 1 Department of Orthopaedic Surgery, Fujita Health University Second Hospital, 3-6-10 Otobashi, Nakagawa-ku, Nagoya, Aichi, Japan; 2 Department of Orthopaedic Surgery, Fujita Health University, 1-98 Dengakugakubo, Kutsukake, Toyoake, Aichi, Japan; 3 Department of Pathology, Fujita Health University, 1-98 Dengakugakubo, Kutsukake, Toyoake, Aichi, Japan

## Abstract

**Introduction:**

Synovitis-acne-pustulosis-hyperostosis-osteitis (SAPHO) syndrome is a rare disorder. The etiology remains unknown and the treatment is still empirical. Synovitis is one of the major manifestations, but information on histopathological features is still lacking. In this case, we investigated the histopathological features of SAPHO syndrome synovitis.

**Case presentation:**

We present the case of a 53-year-old Japanese woman with SAPHO syndrome accompanied by marked knee synovitis and palmoplantar pustulosis. We found abundant sterile joint fluid in the right knee, and a blood test showed abnormally high values of C-reactive protein (17.26 mg/dl) and matrix metalloproteinase-3 (800 ng/ml). Arthroscopic surgery revealed marked proliferation of villous synovial tissues similar to rheumatoid arthritis and standard microscopic findings were also similar to rheumatoid arthritis. Furthermore, for the first time, we demonstrated by immunohistochemistry the expression of tumor necrosis factor-alpha (TNF-α) converting enzyme, TNF-α and matrix metalloproteinase-3 in the proliferated synovial lining cells. After arthroscopic synovectomy, her knee symptoms immediately diminished and laboratory data (matrix metalloproteinase-3 and C-reactive protein) normalized within 2 weeks of surgery.

**Conclusion:**

We demonstrate the expression of TNF-α converting enzyme, TNF-α and matrix metalloproteinase-3 in SAPHO syndrome synovitis for the first time and also show, both macro- and microscopically, the similarity between SAPHO syndrome and rheumatoid arthritis synovitis. These new findings support the recently reported successful treatment of SAPHO syndrome with antirheumatic drugs, especially with anti-TNF-α agents.

## Introduction

Synovitis-acne-pustulosis-hyperostosis-osteitis (SAPHO) syndrome is a rare disorder characterized by osteoarticular and dermatological manifestations, and was first proposed by Chamot *et al.*[1]. The pathogenesis of SAPHO syndrome has not been determined, thus a variety of therapeutic approaches exist. Treatment remains empirical with non-steroidal anti-inflammatory drugs (NSAIDs) and analgesics being the first-line drugs; results are inconsistent and usually inconclusive. Among other drugs, disease modifying anti-rheumatic drugs (DMARDs) such as sulfasalazine and methotrexate are the most frequently employed, but results are also inconsistent. Recently, positive outcomes have been obtained with bisphonates via their anti-osteoclastic effect and anti-inflammatory action, which are related to their suppressive effect on tumor necrosis factor-alpha (TNF-α) [2]. Moreover, some authors have reported the effectiveness of new DMARDs, anti-TNF-α agents, especially for osteoarticular manifestations of SAPHO syndrome [3]-[7]. As has been shown in rheumatoid arthritis (RA), proliferated synovium is a major source of proinflammatory cytokines and proteinases. TNF-α is a key cytokine, which triggers the inflammatory cascade and stimulates the production of matrix degradable proteinases such as matrix metalloproteinases (MMPs) [8]. TNF-α converting enzyme (TACE) processes a membrane form of TNF-α to a soluble form [9], and the binding of the latter form to TNF receptors triggers pathological events in RA. These findings indicate that synovitis and the processing of TNF-α by TACE are very important aspects in the pathogenesis of SAPHO syndrome. However, detailed information on the pathological features of synovitis in SAPHO syndrome is still lacking.

In this report, we describe a patient with SAPHO syndrome accompanied by marked knee synovitis. We demonstrate the expression of TACE, TNF-α and MMP-3 in SAPHO syndrome synovitis for the first time and also show the similarity between SAPHO syndrome and RA synovitis.

## Case presentation

A 53-year-old Japanese woman first presented to a neighborhood clinic in 2001 with palmoplantar pustulosis. In January 2005, the patient experienced lower back pain, and a compression fracture of the fifth lumbar vertebral body was diagnosed at the same clinic. In May 2005, the first episode of painful swelling of her right knee occurred. For the knee pain, conventional therapy with NSAIDs was applied. In June 2006, she presented to our department for the first time with persistent swelling and pain in her right knee, and could not walk without a T-cane (walking stick). Physical examination showed marked patellar ballottement with local heat, and the range of motion was 0–100 degrees. A knee puncture yielded 20 ml of yellow cloudy joint fluid, but cultures for bacteria were negative. At the time, mild, dull lower back pain was still continuing but no other joint pain, including in the costa-sterno-clavicular joint, was found. Palmoplantar pustulosis was observed in both her palms and her soles. Laboratory tests showed elevated indices of inflammation: erythrocyte sedimentation rate 87 mm/hour, C-reactive protein (CRP) level 17.26 mg/dl (normal <0.30 mg/dl) and an abnormally high value of matrix metalloproteinase (MMP)-3 >800 ng/ml (normal range 17.3-59.7 ng/ml). Rheumatoid factor was negative. Radiographic study revealed that the right knee and the lumbar spine seemed to be normal. Magnetic resonance imaging (MRI) revealed knee synovitis and bone marrow edema at the fifth lumbar vertebral body, compatible with sterile inflammation.

**Figure 1:**
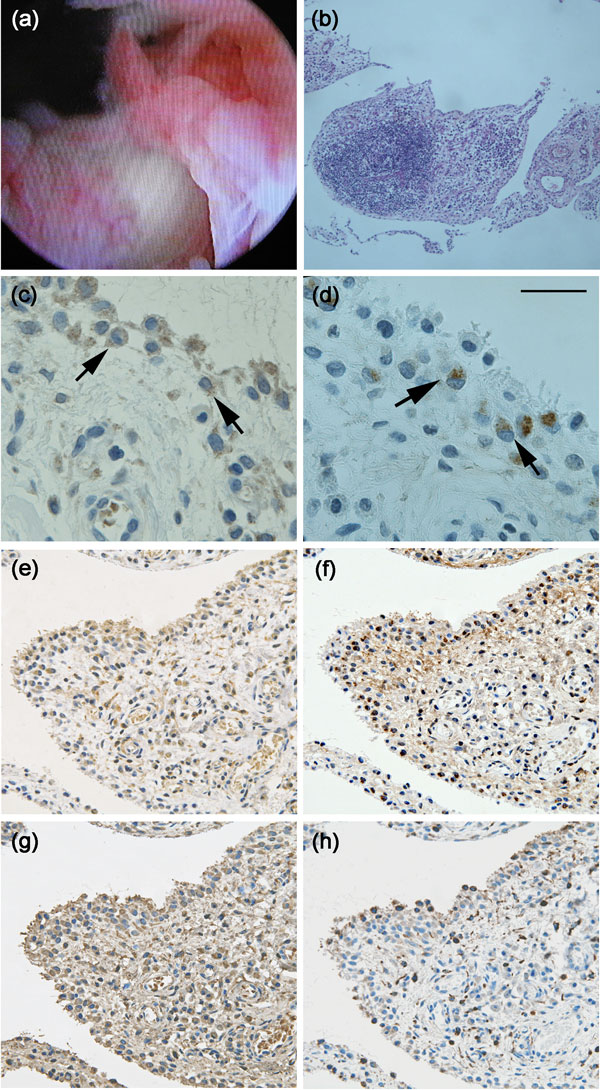
**Macro- and microscopic findings of synovitis-acne-pustulosis-hyperostosis-osteitis syndrome synovitis**. Note that rheumatoid arthritis-like villous contoured synovial tissues with rich blood circulation are markedly proliferated (a). Continuous paraffin sections were stained with hematoxylin and eosin (b) or immunostained with antibodies against tumor necrosis factor-alpha converting enzyme (c, e), matrix metalloproteinase-3 (d, f), tumor necrosis factor-alpha (g), or CD68 (h). Note that hyperplastic synovitis with lymphoid nodules similar to rheumatoid arthritis (b), and tumor necrosis factor-alpha converting enzyme, tumor necrosis factor-alpha and matrix metalloproteinase-3 are expressed in synovial lining cells (c-g). Arrows, synovial lining cells. Scale bar, 100 μm.

In July 2006, arthroscopic surgery was performed for knee synovitis. Intra-operative findings showed marked proliferation of villous contoured synovial tissues with rich blood circulation similar to RA (Figure 1a). Continuous paraffin sections of biopsied synovial tissues were used for histopathological analyses, and standard microscopic study showed hyperplastic synovitis with lymphoid nodules and many blood vessels similar to RA (Figure 1b). Immunohistochemistry revealed the expression of TACE (Figure 1c,1e), TNF-α (Figure 1g) and MMP-3 (Figure 1d,1f) in synovial cells of the lining layer. TACE and TNF-α were expressed dominantly in CD68 positive synovial cells of the superficial lining layer, whereas MMP-3 was expressed in CD68 negative synovial cells of the deep lining layer (Figure 1e-1h). Primary antibodies used for these analyses were polyclonal antibodies for TACE (sc-25782; Santa Cruz Biotechnology, USA), TNF-α (654250; Calbiochem, Germany), monoclonal antibodies for MMP-3 (55-2A2; Daiichi Fine Chemical Co., Japan) and CD68 (M0814; DakoCytomation, Denmark).

After arthroscopic synovectomy, her knee symptoms immediately diminished and laboratory data (MMP-3 and CRP) normalized within 2 weeks of surgery. In the 2-year follow-up period, there was no recurrence of synovitis, no exacerbation of palmoplantar pustulosis, and MMP-3 and CRP levels remained normal.

## Discussion

We investigated the histopathological features of SAPHO syndrome synovitis in our patient. Macroscopic findings showed marked proliferation of villous contoured synovial tissues with rich blood circulation. Microscopic findings showed hyperplastic synovitis with lymphoid nodules and many blood vessels. Further immunohistochemistry showed that TACE and TNF-α were expressed dominantly in CD68-positive macrophage-like synovial cells of the superficial lining layer while MMP-3 was expressed in CD68-negative fibroblast-like synovial cells of the deep lining layer. These histopathological features overlapped with those of RA synovitis. However, the extent of each microscopic feature, such as the hyperplasticity of the lining layers, lymphoid nodules and vascular density, seems to be more marked in RA than in SAPHO syndrome. Although it is difficult to compare our findings with past cases due to lack of reports with detailed histopathological analyses of SAPHO syndrome synovitis, the similarity of the histopathological features between SAPHO syndrome and RA indicates that, at least partially, they have a common synovial pathogenesis.

The treatment of SAPHO syndrome remains empirical, but recently some successful experiences with bisphonates and anti-TNF-α agents have been reported. It is speculated that these drugs act via the suppression of TNF-α action [2]. TACE is a key proteinase in the processing of TNF-α [9] and the processed form can bind to TNF receptors and activate various pathological events including the production of MMPs. In our patient, the highly elevated serum MMP-3 level was normalized after arthroscopic synovectomy. Several studies have indicated that TACE levels are elevated in RA joints compared with osteoarthritis or normal articulations, suggesting that abnormal TACE activity contributes to TNF-α action in RA pathogenesis. The expression of TACE, TNF-α and MMP-3, therefore, encourages us to speculate that TACE plays a role in the pathogenesis of SAPHO syndrome synovitis through the processing of TNF-α, which triggers a cascade of pathological events through a mechanism similar to RA. To the best of our knowledge, 10 cases of SAPHO syndrome treated with anti-TNF-α agents have been described, all of them showing a sustained response on osteoarticular manifestations [3]-[7], but were not favorable for cutaneous manifestations in some cases [5]. The reasons for the negative effects on cutaneous manifestations have not yet been determined. It is premature to exclude anti-TNF-α agents from the therapeutic options for SAPHO syndrome because examples of the application of these agents have been limited to recalcitrant cases. Further investigations are clearly needed to elucidate this rare and complicated disorder and the expression of TACE, TNF-α and MMP-3 in the synovium may provide an important clue for the development of a new therapeutic strategy for SAPHO syndrome.

## Conclusion

We have demonstrated the expression of TACE, TNF-α and MMP-3 in SAPHO syndrome synovitis for the first time and also showed the similarity between SAPHO syndrome and RA synovitis. These new findings support the recently reported successful treatment of osteoarticular manifestations of SAPHO syndrome with anti-TNF-α agents.

## Abbreviations

CRP: C-reactive protein; DMARDs: disease modifying antirheumatic drugs; MMP: matrix metalloproteinase; NSAIDs: non-steroidal anti-inflammatory drugs; RA: rheumatoid arthritis; SAPHO: synovitis-acne-pustulosis-hyperostosis-osteitis; TACE: TNF-α converting enzyme; TNF-α: tumor necrosis factor-alpha.

## Consent

Written informed consent was obtained from the patient for publication of this case report and any accompanying images. A copy of the written consent is available for review by the Editor-in-Chief of this journal.

## Competing interests

The authors declare that they have no competing interests.

## Authors' contributions

KK was the primary physician and orthopedic surgeon, conceived the original study, organized and analyzed the data and prepared the draft of the manuscript. HY, NT and MY were consulting orthopedic surgeons, evaluated laboratory and imaging data, and assisted with manuscript editing. MS and SK collected the patient's information. YM was the pathologist and performed the histological examinations. All authors read and approved the final manuscript.
